# How does the method change what we measure? Comparing virtual reality and text-based surveys for the assessment of moral decisions in traffic dilemmas

**DOI:** 10.1371/journal.pone.0223108

**Published:** 2019-10-09

**Authors:** Leon René Sütfeld, Benedikt V. Ehinger, Peter König, Gordon Pipa

**Affiliations:** Institute of Cognitive Science, Osnabrück University, Osnabrück, Germany; Polish Academy of Sciences, POLAND

## Abstract

The question of how self-driving cars should behave in dilemma situations has recently attracted a lot of attention in science, media and society. A growing number of publications amass insight into the factors underlying the choices we make in such situations, often using forced-choice paradigms closely linked to the trolley dilemma. The methodology used to address these questions, however, varies widely between studies, ranging from fully immersive virtual reality settings to completely text-based surveys. In this paper we compare virtual reality and text-based assessments, analyzing the effect that different factors in the methodology have on decisions and emotional response of participants. We present two studies, comparing a total of six different conditions varying across three dimensions: The level of abstraction, the use of virtual reality, and time-constraints. Our results show that the moral decisions made in this context are not strongly influenced by the assessment, and the compared methods ultimately appear to measure very similar constructs. Furthermore, we add to the pool of evidence on the underlying factors of moral judgment in traffic dilemmas, both in terms of general preferences, i.e., features of the particular situation and potential victims, as well as in terms of individual differences between participants, such as their age and gender.

## Introduction

Ethical considerations concerning autonomous machines and, in particular, self-driving cars have recently gained widespread attention in research, media and society. Questions of trade-offs between utility and safety, liability in the case of accidents, and biases in the detection of ethnic minorities are just some of the open ethical issues brought up by the development of this technology [1–3]. Most prominently, the question how an automated vehicle (AV) should behave in an ethical dilemma situation has been addressed in a large number of publications [4–10]. In these, the problem is typically broken down into a series of forced choice decisions between two options, akin to the trolley dilemma [11], and the decision patterns of participants are analyzed to infer what factors play a role in their decision making, and to which degree. While the purpose of such studies is not to provide a blueprint for the behavior of self-driving cars, their findings can inform the debate, point out where our intuitive moral judgment is at odds with moral theories and regulations, and deliver initial numerical values for formal decision making models [12]. On the regulatory side, a first advance towards defining a legal framework for the use of AVs was undertaken by an ethics commission of the German Federal Ministry of Transport and Digital Infrastructure [13]. With respect to decisions in dilemma situations, the commission precludes the consideration of individual features such as age or gender, but remains inconclusive about the consideration of the number of people harmed in any given option. The report also doesn’t offer concrete suggestions for the design, implementation, or regulation of ethical decision making systems, citing a need for more research. There is consensus, however, that the systems need to be transparent, and the rules they obey to be suitably communicated to ensure public acceptance of AVs.

While it remains debatable which factors should be taken into account to make a decision in dilemma situations, a frame of reference can be derived from human decision making. The MIT’s Moral Machine is a large-scale survey analyzing various factors that influence our moral decisions in dilemma situations, distinguishing between features of the situation (termed global preferences), and individual variations between the participants [10]. In the analysis of the global preferences, the largest effects were found for favoring humans over pets, larger groups of characters over smaller ones, and younger people over older ones. Further notable effects were found favoring those who behave lawfully, those with a higher social status, the physically fit, females over males, and pedestrians over passengers of the AV. A small effect was also found favoring inaction over action, suggesting that some reluctance to interfere in such a situation is part of our moral intuitions, but is often outweighed by utilitarian considerations. On the side of individual variations among the participants, small effects were found, for instance, for the participants’ age and gender. Many of these findings are qualitatively corroborated by other studies. For example, [6–9] all found strong tendencies towards favoring larger groups and younger people, and [5] previously found strong effects towards favoring humans over animals. Thus, studies report a variety of factors that influence human decision making in dilemma situations.

Interestingly, we observed a large variety in the approaches used to assess the factors of human decision making. The Moral Machine, for example, is a web browser-based survey using simple birds-eye view drawings of the scenarios in question [10]. By contrast, [5–7] used interactive virtual reality (VR) applications, showing the scenarios from the driver’s perspective. Here, the decisions had to be made in real time, with response time windows of four seconds in most cases. [8] placed participants in a driving simulator, also presenting the scenarios from an immersive first person view, but freezing the scene at decision time and supplying additional information about the situation using text-overlays. [4, 9, 14], on the other hand, used predominantly or entirely text-based surveys in their studies. The large differences between these approaches raises the question, to what extent the same underlying construct is measured. In fact, we know from studies in the field of empirical ethics that contextual factors and the way we frame the question can have a sizable impact on the ethical decisions we make [15–17]. The large discrepancies between moral decisions in VR and text-based assessments, found in [18, 19] even suggest that moral judgment and moral action may be distinct constructs. However, the thought experiments used in empirical ethics are usually constructed to emphasize a clash of different moral schools of thought—typically deontologism, focused on moral rules that must not be broken (“do not actively kill another person”), and utilitarianism, focused on minimizing overall harm, thus saving the largest amount of people. Unlike most classical dilemma thought experiments, traffic dilemmas are not typically designed to emphasize a clash of different moral intuitions. Instead, they are usually aimed at the participants’ evaluation of the potential victims and the environmental circumstances. The context of traffic scenarios is also arguably closer to most people’s day-to-day reality, possibly making it easier to fall back on existing evaluations or behavioral instincts. To what degree the methodology of assessment has an influence on the decision patterns in traffic dilemmas, thus, remains an open and very relevant question that we address in the present work.

### Research objective

In this paper, we analyze how the participants’ behavior and decisions are influenced by the presentation of traffic dilemmas. The approaches used in the literature often vary in multiple aspects, making it difficult to trace how these aspects influence the participants’ behavior. These aspects of the assessment can, to a large extent, be broken down into the level of abstraction, the presentation modality (desktop vs. virtual reality), and varying degrees of time pressure. To isolate the corresponding effects, we designed two traffic dilemma studies, in which we systematically vary the presentation of the dilemmas across these three dimensions. All in all, the two studies cover a spectrum of presentation styles from text-based questionnaires to a fully immersive VR experience akin to [5]. The effects of time pressure on the decision making process were examined, since some form of time limitation is an inherent aspect of decision making in immersive VR. Ultimately, this establishes how the different approaches used in the literature relate to each other, and it can inform us about potential biases in the participants’ moral judgment connected to the assessment methodology.

In our statistical models, we also included personal features of the participants as predictive factors of behavior. These include the participants’ age and gender, as well as two more variables of particular interest in this context: Video game experience and social desirability. Video game experience was included as a potentially explanatory variable, since virtual reality studies are arguably similar to video games in terms of visual and acoustic presentation, as well as user input. Frequent video game players might, therefore, have a different perception of the stakes involved in their decisions. Social desirability, i.e., a tendency in participants to answer in accordance with social norms instead of their true beliefs, might lead to systematic shifts in the decision patterns, so we assessed this tendency with the social desirability scale (SDS-17) questionnaire [20], and incorporated the respective scores as factors in the analysis.

## Methods

### Study 1

**Fig 1:**
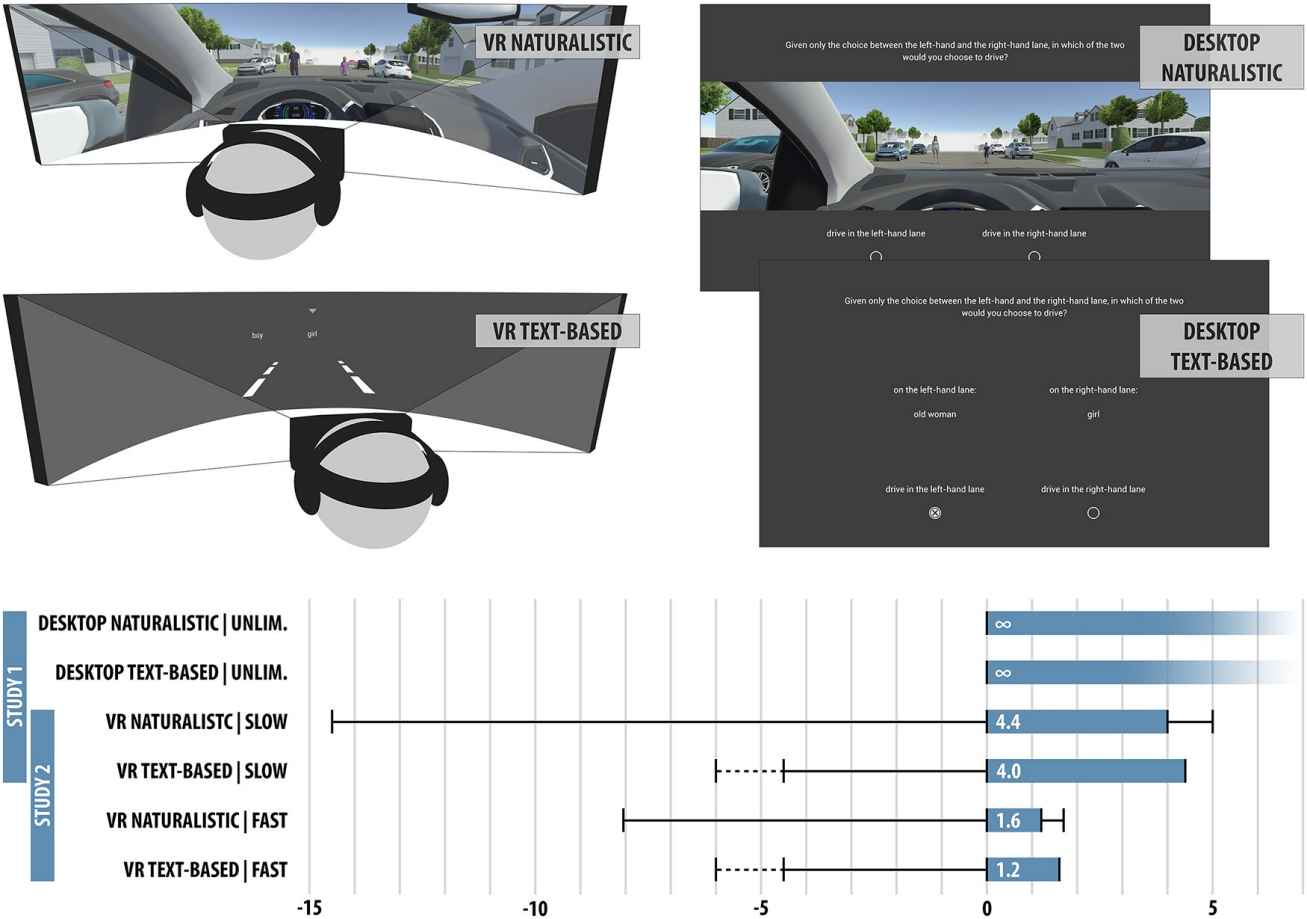
Overview of the experimental conditions and time lines. Black T-lines on the left indicate trial and control onsets, dashed lines indicate variable onset times. Blue bars indicate response time windows from visibility onset of the obstacles (VR: appearing from the fog) until car control offset, and black T-lines on the right indicate the time the car kept moving after control offset, i.e., the end of the trial. VR conditions featured an additional 1.5s of fade-to-black time (not indicated in the graphic).

In the first study, we employed a 2x2 experimental design with the factors level of abstraction (naturalistic vs. text-based; within subjects) and presentation modality (VR vs. 2D desktop monitor; between subjects). *Levels of abstraction*: The naturalistic settings featured a rendered 3D environment, showing the dilemma situation from the driver’s first-person view (see Fig 1). By contrast, the text-based settings replaced the 3D environment with text and simple visual indicators on a gray background. *Modality*: In both the naturalistic VR and text-based VR settings, participants wore a head-mounted display (HTC Vive) and headphones, allowing them to freely look around the respective environment. They had to make their decision within 4.0s (naturalistic) and 4.4s (text-based), respectively. In the desktop modality, participants were presented with a fixed screen, and the time to make a decision was unlimited in both naturalistic and text-based conditions. For a more detailed description of the experimental conditions, please refer to S1 Appendix.

The four conditions spanned a spectrum from *questionnaire-like* (text-based desktop) to *as realistic as possible* (naturalistic VR), while allowing us to treat the level of abstraction and the modality as separate factors in the analysis. We recruited 88 participants, mostly from the local university, and had to exclude three due to misunderstanding the instructions or crashes of the application. The remaining 85 participants (43 females, 42 males, mean age 23.0, for further information see S1 Table) were randomly assigned to either the VR (43) or desktop (42) condition, and reported their age and gender in the application before the trials started. In the experiment, they were presented with a block of 20 trials in the naturalistic setting, then with a block of 20 trials in the text-based setting, or vice versa (order assigned randomly). In each trial, participants chose which of two single obstacles on the road ahead of them they would rather spare, with the obstacles being randomly drawn from a pool of animals (dog, goat, and boar), and humans of different gender and age (young, adult, or elderly). Additionally, some trials featured an empty lane, as a form of sanity check.

### Study 2

In the second study, we again employed a 2x2 experimental design, this time with the factors level of abstraction (naturalistic vs. text-based; within subjects) and speed (slow vs. fast; within subjects). The slow condition was identical to study 1, the fast conditions had smaller response time windows of 1.2s (naturalistic) and 1.6s (text-based). Fast response times in the naturalistic setting were achieved by increasing the car’s speed and decreasing the viewing distance. All conditions used the VR environment as described in study 1. The experimental procedure was divided into blocks by level of abstraction, showing at random either both naturalistic or both text-based conditions in a row. The order of the condition (speed) within these blocks was randomized. Each condition consisted of 7 trials, which were largely identical to those in the first study, except that animals were excluded from the obstacle pool to get a larger number of human vs. human trials. We recruited 107 participants, but had to exclude 14 due to an error in the application. Of the remaining 93 participants, 58 were females, and 35 males (mean age 21.3, for further information see S1 Table). Subjects reported their gender and age before the main experiment, filled in a short post-hoc questionnaire, as well as paper-based version of the SDS-17 questionnaire [20] (an assessment of their social desirability) after finishing the experimental trials.

Both studies conformed to the Code of Ethics of the American Psychological Association, as well as to national guidelines, and were approved by the Osnabrück University’s ethics committee. A more detailed description of the conditions and controls can be found in S1 Appendix. The used hardware and the precise experimental timelines are defined in S2 Appendix.

### Statistical modelling

For the behavioral analysis, we employ Bayesian hierarchical logistic regression models to predict which lane a participant would choose, based on a number of explanatory variables characterizing the trial.

In its basic form, logistic regression models the probability of the outcome of a binary dependent variable. That is, the model finds the set of parameters that jointly determine the probability of finding a positive or negative outcome in a specific trial, maximizing the likelihood of observing the experimental data as they are. With some simplifications, one can interpret the parameters estimated by the model as modifiers of an object’s ethical valuation. Positive log-odds thus indicate higher ethical valuation of the feature in question, negative log-odds a lower valuation.

In Bayesian statistics, we begin the modelling process with prior distributions, expressing our knowledge or belief about the impact of the modeled variables before seeing the data. In the model fitting step, the variables, or model parameters, are then approximated to realize a compromise between the chosen priors and the best fit for the observations made. The resulting distributions, called posterior distributions, ultimately represent the knowledge we have about the model parameters after seeing the data, with the posterior mean representing our best guess for the true impact of a given variable. In this analysis, we used weakly regularizing priors, representing a prior believe that the variables do not take on extreme values (see S4 Appendix).

In this framework, no classical significance tests are performed. Instead, the evidence is treated as being on a continuous scale. The sign and magnitude of a parameter tell us about the direction and size of an effect, while the credibility intervals tell us how certain we can be that it is different from zero. Additionally, the Bayes factor provides a measure for how much our knowledge about a given parameter changed from the prior, based on the observations we made. Bayes factors between $\frac{1}{3}$ and 3 are generally regarded as inconclusive, with anything below $\frac{1}{3}$ being regarded as evidence against the null hypothesis (the hypothesis that the parameter has no influence on the outcome), and anything above 3 regarded evidence in favor of the null hypothesis.

The variables we used in the model can be divided into three categories: (1) Global preferences, such as age or gender of the potential victims, (2) features of the assessment, i.e., modality, abstraction and response time, and (3) individual features of the participants, such as their age or SDS-17 score. For all models, we used the maximal multi-level model [21], similar to the Bradley-Terry-Luce model of paired comparisons [22], with parameters for individual subjects on the second level. We fitted one model per study, and both models made use of the features of the portrayed situation and features of the assessment. In study two, the larger number of relevant trials allowed us to further include the individual subjects’ features. Besides the main effects, we restricted the interactions considered in the model. For study 1, we modeled only interactions between global preferences, abstraction and modality. In study 2, we modeled interactions between global preferences, abstraction and speed, as well as between global preferences and each of the individual participant’s factors. The model specifications and the chosen weakly informative priors are laid out in more detail in S4 Appendix.

## Results

**Fig 2:**
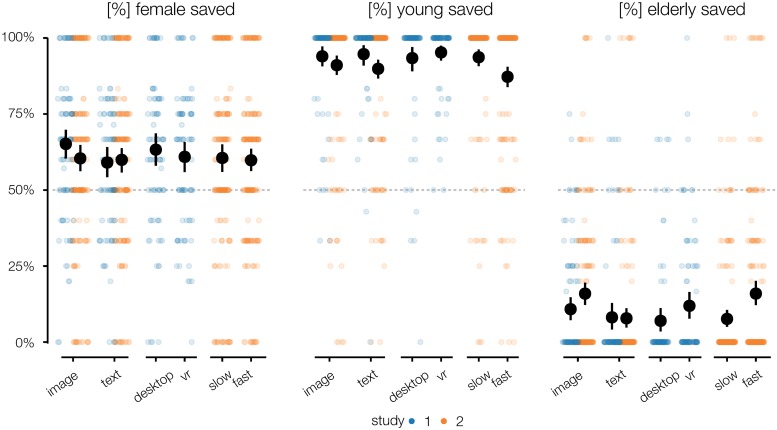
Saving rates. Rates of saving young (left), male (middle) and elderly (right) people, by study and experimental condition.

The absolute rates of saving obstacles of different age groups and genders in both studies are provided in Fig 2. This descriptive view shows us that across conditions, females were saved in about 60% of all cases, children were saved in about 90% of all cases, and the elderly were saved in about 10% of all cases. Since the differences between the experimental conditions are small in comparison to these results, we can already infer that the potential victims’ age and gender were dominant factors in the participants’ decisions.

### Global preferences

**Fig 3:**
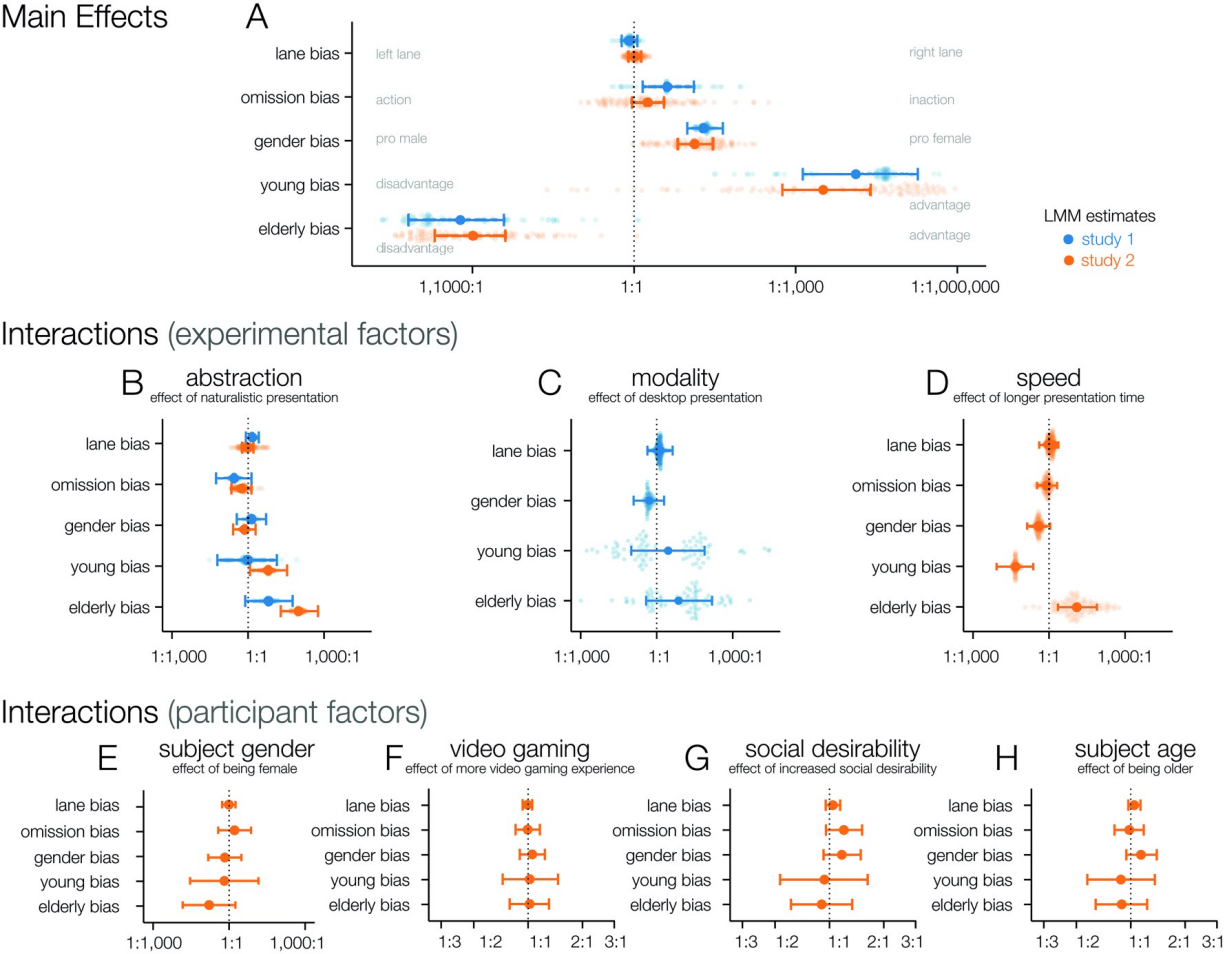
Bayesian hierarchical logistic regression results. A) Main effects of a logistic linear mixed regression model for both studies. Error bars indicate 95% credible intervals around the median posterior estimate of the group estimate. Dots indicate estimations of the individual participants’ parameters. B-D) Interactions of experimentally controlled factors. E-H) Between subject factors, note the different scale for F-H and that these are slopes (effects per unit) and not categorical effects.

For a detailed assessment of all involved factors in the decision making process, we used a Bayesian logistic regression model analysis. We refer to features of the potential victims, such as their gender and age, as well as the lane they are in, and the lane the participants’ car is in at the onset of the obstacle, as global preferences. The magnitude of influence these features have on the outcome of a trial for both studies are shown in Fig 3, and corresponding tables can be found in S5 Appendix. *Lane bias*: A lane bias describes a tendency to prefer either the right or the left lane, irrespective of the obstacles in those lanes. The mean a posteriori log-odds estimates for this are very close to 0, with the posterior distribution carrying a lot of weight on either side of it and the Bayes factors strongly preferring no bias (study 1: −0.2, *CI*_95_: −0.5, 0.2, *BF*_*H*0_: 11.1; study 2: 0.1, *CI*_95_: −0.2, 0.3, *BF*_*H*0_: 21.3). Thus, while it would be plausible that a right-hand lane bias exists due to right-hand traffic in Germany, any general lane bias in this sample is minimal at best and would not have any notable effect on their decisions. *Omission bias*: An omission bias shows a general tendency towards inaction, which may be rooted in an aversion to active causation of harm. This bias was observed in study 1 (1.4, *CI*_95_: 0.4, 2.6, *BF*_*H*0_: 0.1) and inconclusive in study 2 (0.6, *CI*_95_: −0.1, 1.3, *BF*_*H*0_: 1.7). However, even in study 1 its size is small in comparison to the effects of gender and age, and played only a subordinate role in the participants’ decisions. *Gender bias*: A considerable bias in favor of female obstacles was observed in both studies (study 1: 3.0, *CI*_95_: 2.3, 3.8, *BF*_*H*0_: 0.0; study 2: 2.6, *CI*_95_: 1.9, 3.4, *BF*_*H*0_: 0.0). The small random effects standard deviations for the estimated individual parameters (study 1: 0.7, *CI*_95_: 0.03, 1.87, study 2: 0.45, *CI*_95_:0.97, 2.73) also indicate a high consensus within the sample population. *Age bias*: The age of the potential victims had the largest influence of all considered factors on the trials’ outcomes, with mean posterior estimates of 9.5 (young) and −7.4 (elderly) in study 1, and 8.1 (young) and −6.9 (elderly) in study 2. Interestingly, the between-subjects variance of the age bias is fairly large, indicating a weaker consensus about the extent of the age bias in the sample population (study 1: 3.9, *CI*_95_: 0.1, 7.4, study 2: 3.4, *CI*_95_: 2.1, 5.0). Overall, these findings are in line with the existing literature [5, 7, 10], supporting the general suitability of this paradigm to test influences of the assessment methodology on the participants’ behavior.

### Features of the assessment

We turn now to the effects of the assessment methodology on the behavior, shown in Fig 3. These effects are to be interpreted as interactions on, or modifiers of the global preferences presented in the previous paragraph, and tell us how the behavior changes if we change the respective aspect of the assessment.

#### Level of abstraction

Going from a text-based to a naturalistic presentation showed no conclusive effect on the omission bias (study 1: −1.3, *CI*_95_: −2.9, 0.3, *BF*_*H*0_: 1.1; study 2: −0.6, *CI*_95_: −1.5, 0.3, *BF*_*H*0_: 2.9), but strong evidence for higher valuation of elderly people in study 2 (study 1: 1.8, *CI*_95_: −0.3, 4.0, *BF*_*H*0_: 0.7; study 2: 4.6, *CI*_95_: 3.0, 6.3, *BF*_*H*0_: 0.0). To determine whether the inclusion of the level of abstraction as a predictor improves our model fit, we used the brms package [23, 24] to calculate the difference in Watanabe–Akaike Information Criterion (WAIC) between the full model, and an identical model without any abstraction-related fixed effects (using the same random structure). Study 1 shows slight evidence against the more complex model (Table 1). Since the standard error of the difference is small compared to the magnitude of the difference, the simpler model without level of abstraction as a factor is the superior model here. Study 2, on the other hand, shows slight evidence in favor of the more complex model (Table 1). However, the magnitude of the difference is smaller than in the first study, and the standard error of the difference is larger, giving more credibility to the findings from study 1. Moreover, the observed influence of level of abstraction as a whole is likely driven by the interaction between level of abstraction and elderly bias. We therefore conclude that the level of abstraction has no significant influence on the participants’ decisions outside of the valuation of the elderly.

**Table 1:** WAIC model comparison: Level of abstraction.

	study 1		study 2	
parameter	WAIC	SE	WAIC	SE
with Abstraction	598.4	35.5	1141.85	50.1
without Abstraction	570.2	35.6	1118.3	51.2
with—without	28.2	5.77	-23.6	10.5

Model with fixed effects of level of abstraction against model without them, for both studies individually.

#### Modality

Contrasting an immersive VR environment to a desktop setting with static scenes and unlimited response times did not yield any conclusive effects, except strong evidence that the lane bias is independent of the modality (0.2, *CI*_95_: −0.5, 0.8, *BF*_*H*0_: 8.3). We can further exclude large effects of modality on the gender bias (−0.7, *CI*_95_: −2.1, 0.7, *BF*_*H*0_: 2.7). For the young and elderly interaction we observe a bimodal distribution in our data (see Fig 3), which leads to large credibility intervals of the effect for the interaction of young (1.0, *CI*_95_: −2.3, 4.4, *BF*_*H*0_: 1.5) and elderly (2.0, *CI*_95_: −0.9, 5.0, *BF*_*H*0_: 0.8).

#### Time pressure

High time pressure, on the other hand, did have a considerable impact on the decision patterns, as it led to systematic decreases of the age bias (young: −3.0, *CI*_95_: −4.8, −1.4, *BF*_*H*0_: 0.0; elderly: 2.5, *CI*_95_: 0.8, 4.3, *BF*_*H*0_: 0.1) as well as a trend towards lower gender bias (−1.0, *CI*_95_: −2.0, 0.1, *BF*_*H*0_: 1.1), but with an inconclusive Bayes factor. Overall, this result is in line with more randomness in the participants’ answers, or in other words, an increased error rate. However, it was not caused by a simple failure to elicit a response in time. Running out of time to enter a response would lead to fewer lane changes overall and cause an increased omission bias, both of which we did not observe (omission bias: −0.2, *CI*_95_: −1.1, 0.7, *BF*_*H*0_: 5.9; lane changes see S3 Appendix). However, we cannot discern whether, or to which extent, this systematic effect is the result of errors, such as pressing the wrong button or misidentifying an obstacle’s gender or age under time pressure, or the result of interrupting the cognitive process of evaluating ethical aspects of the situation. Mind that these effects don’t seem to carry over to the slower VR conditions with 4.0-4.4s response time windows. The absence of systematic effects between time-constrained and unconstrained modalities, and the fact that 72% (text-based) to 82% (naturalistic) of all responses in the unconstrained settings were made within the response time windows of the time-constrained settings (see S3 Appendix), suggest that response time windows of about 4.0s are not restricting the validity of immersive VR-based assessment. We conclude that aside from very high time pressure, the observed decisions are remarkably consistent across different approaches to the experimental assessment.

### Individual features of the participants

This third set of features refers to individual differences between the participants, namely their age and gender, as well as video gaming experience and susceptibility to social desirability. The influence of these features on the behavior is again modeled as interactions with the global preferences, to be interpreted as deltas on them.

#### Gender

Female participants showed a tendency to value elderly people higher than male participants did (−1.8, *CI*_95_: −4.2, 0.6, *BF*_*H*0_: 0.8) but the Bayes factor is inconclusive. Interestingly, we found (weak) evidence for no effect between male and female participants with respect to gender bias (−0.4, *CI*_95_: −1.9, 1.1, *BF*_*H*0_: 3.6), supporting the notion that a pro-female gender bias is generally agreed upon in the sample population.

#### Age

We modeled the influence of age using the continuous age predictor with several interactions, and the resulting parameter estimate is to be interpreted as change in odds per year. We found evidence against any effect of participants age (all *BF*_*H*0_:>12).

#### Video game experience

The amount of video game experience (measured in game playing hours per week) had virtually no influence on any of the parameters, rejecting the hypothesis that frequent players could have a different conception of the stakes involved in these scenarios (all *BF*_*H*0_:> 17).

#### Social desirability

Higher scores in the SDS-17 characterize an increased tendency to respond in line with social norms instead of one’s own true beliefs. We found evidence against any moderate or large effects of SDS-17 scores (all *BF*_*H*0_:> 7), but the credible intervals indicated trends towards small effects in omission bias (0.18, *CI*_95_: −0.05, 0.42, *BF*_*H*0_: 7.3) and pro-female gender bias (0.16, *CI*_95_: −0.08, 0.40, *BF*_*H*0_: 10.4). People with a stronger tendency to be influenced by social norms may thus prefer not to take action, in order not to increase their perceived own guilt, and a higher valuation of females would arguably be in line with social norms in modern western societies. However, our analysis makes anything but small effects in terms of omission and gender bias unlikely.

## Discussion

We conducted two studies to show whether different assessment methods change the ethical decisions of participants in road traffic dilemma situations. Our main finding is that by and large this seems not the be the case, and VR and text-based assessments appear to measure the same underlying construct, with only minor shifts in behavior between the different methods. By extension, this supports the suitability of the methodology and comparability of the results obtained in previous studies [5–10].

Personal involvement has previously been linked to “reduce[d] sensitivity to moral norms and [an] increase[d] general preference for inaction” [25]. In an exploratory post-hoc questionnaire in study 2, participants reported they could more easily put themselves in the presented situation in the naturalistic trials, than in the text-based ones (naturalistic: 5.1/7; text-based: 4.2/7). This would lead to the assumption that the omission bias should increase in naturalistic settings. On the other hand, taking the driver’s perspective in a naturalistic setting may cause participants to perceive both options as actively causing harm, thus taking away the supposed moral superiority of inaction. However, while the sign of the posterior means indicated a slight reduction in omission bias in both studies, our results were inconclusive with regard to this question. The most pronounced difference between the naturalistic and text-based representations was a higher valuation of the elderly in naturalistic settings. It is possible that the abstract, text-based representation of “elderly” strips the human person of all other attributes, making age more salient than it would otherwise be. This effect of age could, therefore, be an artifact of the abstract presentation. Some participants, however, reported needing longer to distinguish between elderly and adults in the naturalistic environment than in the text-based setting. The observed effect could, thus, also be an effect of the elderly’s particular visual representation in the virtual environment. Participants may have perceived them closer in age to adults, resulting in a higher valuation. If a naturalistic environment led to a generally reduced influence of the victim’s age on the decisions, we would expect the value of young people to decrease in this setting, which it did not.

The level of abstraction in the presentation was experimentally detached from its modality, i.e., whether the decisions were made in a VR environment under time-constraints, or in a regular desktop setting without any time-constraints. While we can exclude large effects of modality on gender bias, our findings on the omission and age biases were inconclusive.

Severe time pressure, i.e., response time windows of 1.2 and 1.6 seconds, respectively, caused a systematic decrease of gender and age biases, consistent with the notion of erroneous identification of the potential victims, or interruption of the cognitive evaluation of the situation. However, no such effects were found between conditions of 4.0 second and unlimited response windows, suggesting that response windows of about 4 seconds are not long enough to have a considerable impact on the participant’s decisions in the presented scenarios. Time constraints have previously been found to influence moral judgments in some cases [26], but not in others [27, 28]. When found, such differences are typically viewed as evidence in favor of the dual process theory, which links fast and intuitive cognitive processes to deontological reasoning, and slower cognitive processing to utilitarian reasoning [29]. If we interpret deliberate inaction as a deontological choice, in which the norm of not actively causing harm trumps a higher perceived valuation of the obstacle in the given lane, we could construe the lack of a difference in omission bias between the fast and slow condition as evidence against the dual process theory. However, such an interpretation is difficult for two reasons. (1) Equating inaction with deontological judgment is problematic, since the two may generally represent independent factors in moral decision making [25]. (2) Even if we allow this equation, it is not clear whether the omission of a lane-change would be perceived by the participants as refraining from active interference, since the active operation of the car may negate this notion.

What does this mean for future studies? The assessment of more complex and dynamic traffic situations could benefit from the use of VR, in cases where the experimental situations become difficult to fully or precisely explain in text or still images. At the same time, VR assessment is rather costly, requiring specialized hardware and substantial development time to create the applications. The assessment itself is also cumbersome in comparison to abstract, possibly browser-based surveys. The relatively low cost and the ease of reaching large numbers of participants, as exemplified in the Moral Machine [10], make simple and abstract presentations of the scenario the economically preferred choice.

Outside of the examination of different approaches to the assessment, our findings largely support earlier studies [5–10], forming a coherent picture of the ethical principles in our society, as they apply to traffic dilemmas. Unsurprisingly, animals are generally valued far inferior to humans. When having to decide between multiple potential human victims, the utilitarian principle of minimizing the overall harm appears to outweigh most other factors, while a tendency towards inaction only plays a minor role in the decisions. Beyond these, the potential victim’s age appears to be the most decisive factor, followed by their gender. A quantitative comparison between the different studies is, unfortunately, difficult to obtain, due to different experimental setups and modeling approaches. Notably, the observed differentiation by age, gender, and other personal factors stands in contrast to the ethical rules outlined in the report of the ethics commission of the German Federal Ministry of Transport and Digital Infrastructure [13]. In their report, sacrificing an innocent person for the greater good is viewed as strictly unacceptable, and even basing a decision on the number of lives saved between already involved parties is met with severe ambivalence. The findings in this and previous studies thus highlight severe points of contention that need to be addressed by manufacturers and legislators, since they may affect public acceptance of automated driving technology.

With respect to personal factors influencing the decisions, we found no difference in the pro-female gender bias between male and female participants, attesting a high consensus on this aspect. This finding is at odds with the findings of the Moral Machine, where female participants were found to have a much stronger pro-female gender bias than males [10]. This difference may be attributed to the non-representative sample on our side, consisting mostly of undergraduate university students.

We further found females to value elderly people higher than males did, thus having a smaller overall age bias, while the Moral Machine found female respondents to have a slightly larger age bias than males. A possible explanation for this discrepancy lies in the use of a single scale for age bias in the Moral Machine, which could be masking the differential valuation of individual age groups we report. Beyond this, we found no notable effects of the age of the respondents or their experience with video games. A factor that has not been accounted for in previous traffic dilemma studies is that of social desirability. We found marginal evidence for higher scores on the Social Desirability Scale (SDS-17) predicting larger omission and gender biases, creating a leverage point for future studies in this field.

### Outlook

Future studies could address a factor that was only partially discussed in earlier work: When participating in road traffic, be it as a pedestrian, cyclist, or car driver, we consent to a certain level of risk depending on our actions. For instance, common sense dictates that the safety of pedestrians on the sidewalk takes precedence over pedestrians stepping into the street or even jaywalking. This introduces the aspect of fairness to the question, which trajectory to select or whom to put at risk in a critical situation. Since the written law typically does not provide a nuanced conception of consent and fairness, the topic is a prime candidate for empirical assessments. Aside from considerations of fairness, the individual options in dilemma situations will often have different levels of expected collision severity, or expected speeds at impact. This might substantially influence one’s moral assessment of a given situation, but hasn’t been systematically addressed in traffic dilemma studies so far. We believe that these aspects could provide valuable insight into our moral intuitions as they relate to road traffic and possible solutions for ethical decision making in self-driving cars.

## Conclusion

In conclusion, the present work establishes the general comparability of trolley-like traffic dilemma studies using various methods of assessment. It further substantiates previous findings on global preferences guiding our decisions in these scenarios, helping to inform regulation, communication, and possibly implementation of ethically sound decision-making systems in self-driving cars.

## Supporting information

S1 Table Sample overview. (PDF)

S1 Appendix Conditions and controls. (PDF)

S2 Appendix Timelines and hardware. (PDF)

S3 Appendix Trials and error rates. (PDF)

S4 Appendix Bayesian logistic regression model specifications. (PDF)

S5 Appendix Regression coefficients. (PDF)
