# Supplementary material for: How does the method change what we measure? Comparing virtual reality and text-based surveys for the assessment of moral decisions in traffic dilemmas
Source: PLoS One. 2019 Oct 9;14(10):e0223108. doi: 10.1371/journal.pone.0223108 (PMC6785059; doi:10.1371/journal.pone.0223108)
Supplement: S1 Table — (PDF) [file pone.0223108.s001.pdf]

	study 1	study 2
participants recruited	88	107
excluded from analysis	3	14
remaining	85	93
females	43 (50.6%)	58 (62.4%)
males	42 (49.4%)	35 (37.6%)
age	23.0 (SD 3.6; 18-40)	21.3 (SD 3.8; 18-47)
using German language	85 (100%)	88 (94.6%)
using English language	0 (0%)	5 (5.4%)
VR setting	43 (50.6%)	93 (100%)
desktop setting	42 (49.4%)	-
selection criteria	aged 18+	
	no prior participation in similar studies	
	no traumatic traffic-related experiences	
	no epilepsy or psychotic disorders	
further information	mostly university students (cognitive sc., psychology)	

Table 1. Sample descriptions for both studies.

Supporting information

S1 Table: Sample overview.
