# Supplementary material for: How does the method change what we measure? Comparing virtual reality and text-based surveys for the assessment of moral decisions in traffic dilemmas
Source: PLoS One. 2019 Oct 9;14(10):e0223108. doi: 10.1371/journal.pone.0223108 (PMC6785059; doi:10.1371/journal.pone.0223108)
Supplement: S1 Appendix — (PDF) [file pone.0223108.s002.pdf]

Supporting information

S1 Appendix: Conditions and controls. For the naturalistic VR setting, we used a custom 3D environment portraying a suburban residential area. The participants were virtually placed in the driver's seat of a compact car, heading down the road at 36 kph (≈22 mph), and wore headphones playing engine, road and wind noises, as well as a tire rubbing sound during lane changes. The car's starting lane was pseudo-randomly assigned. A wall of dense fog was placed at a fixed distance of about 50m to the car, such that after the obstacles appeared from the fog, participants had exactly 4.0s to decide which lane they would choose, and no braking or other ways of preventing a collision were possible. Inputs were made using the keyboard's arrow keys, and the time remaining to make a decision was indicated acoustically by a series of four beeps, the last one of which had a higher pitch and marked the end of the decision time frame. At this point, control over the car was taken from the participants, and the car went on for another 10 meters in order to complete any ongoing lane changes. The car stopped abruptly right before impact with the obstacles, at which point all sound was muted and the screen faded to black, marking the end of the trial.

In the text-based VR setting, the immersive 3D environment was replaced by an abstract visualization of the same scenario. The chosen lane was indicated by white lane markings on an otherwise uniformly gray background, and the obstacles were indicated as white text. The obstacle currently at risk was positioned centrally above the lane markings and was additionally marked with a small arrow, while the other obstacle was placed to its left or right. Lane changes were indicated by shifting the arrow to the other obstacle, and moving the obstacle texts such that the new obstacle at risk would again end up centered above the road markings. The decision time in this condition was set to 4.4s. The additional 0.4s over the naturalistic condition served to make up for the longer time required to read and comprehend the situation in text-based settings, with the aim of aligning the true decision time and perceived time pressure between the conditions. The precise value was estimated and agreed upon in a pre-study assessment with four participants. The same auditory cue as in the naturalistic condition was used to indicate how much time was left to make a decision. Additionally, the lane markings were progressively covered up over the course of the 4.4s, resembling a declining progress bar.

In the desktop conditions, participants were presented with a questionnaire that either used a still 3D rendering (naturalistic), or a text-based description of the situation. In both cases, participants were asked the same question: "Given only the choice between the left-hand and the right-hand lane, in which of the two would you choose to drive?" In contrast to the VR conditions, the desktop conditions had no default answer, but showed two open circles to select from. The selection was done via the left and right arrow keys and could be changed back and forth until it was confirmed by pressing the space bar. Importantly, there was no default option, and no time limits were imposed in the desktop conditions. While this design decision did not allow us to fully disentangle the influence of the modality from that of time pressure, it did allow us to examine the response times in the absence of any time pressure, allowing us to gage the amount of time pressure imposed with a four second time limit. Moreover, an unlimited time frame puts these conditions more in line with browser-based questionnaires used in the literature.

The slow conditions in the second study were identical to those in the first study, while the fast conditions had the reaction time window reduced to 1.2 and 1.6 seconds for the naturalistic and text-based conditions, respectively, maintaining the 0.4 seconds

of extra time for the text-based settings. In the naturalistic condition, this was achieved by increasing the car's velocity twofold and shortening the fog distance accordingly. In the text-based condition, all visual indicators were sped up to match the shortened time frame. The acoustic indicators (a series of four beeps) were also adjusted accordingly in both conditions.
