# Supplementary material for: How does the method change what we measure? Comparing virtual reality and text-based surveys for the assessment of moral decisions in traffic dilemmas
Source: PLoS One. 2019 Oct 9;14(10):e0223108. doi: 10.1371/journal.pone.0223108 (PMC6785059; doi:10.1371/journal.pone.0223108)
Supplement: S2 Appendix — (PDF) [file pone.0223108.s003.pdf]

Supporting information

S2 Appendix: Timelines and hardware. **Study 1.** Prior to the experiment, the subjects were informed that they had the option to stop the experiment at any time, that their decisions would not be monitored in any way and would be saved anonymously, and signed a consent form. Participants in the desktop screen setting sat in front of a 24” PC screen, whereas in the VR setting, participants wore an HMD (HTC Vive), as well as a pair of headphones (Sennheiser 380 Pro or Bose QuietComfort 25). Regular PC keyboards were used for all user inputs, and participants wore electrodes of an EDA measuring device on two fingers of the left hand. The participants were asked to place their right hand’s fingers on the arrow keys and their left hand’s fingers on the space bar, since no other keys had to be used in the experiment. In the application, the experimental trials were preceded by a form asking for the participant’s age and gender, and a text explaining the controls and introducing the upcoming experimental condition. To give participants in the VR setting a chance to familiarize themselves with the virtual environments, test trials were conducted before each of the two main experimental conditions (naturalistic and text-based), where a number of traffic cones had to be avoided. When a traffic cone was hit, the training trial was repeated until all cones were cleared in a single run. The total time spent in the experiment was about 12 to 15 minutes for participants in the VR setting, and about 5 minutes for those in the desktop screen setting. After completion of all trials, the participants were interviewed about the immersion in the different scenarios and the correct apprehension of the presented task, using standardized questions.

Study 2. The procedure for study 2 was mostly identical to the VR setting in study 1. The total time participants spent in the application was also between 12 and 15 minutes. An exploratory post-hoc questionnaire with questions about the apprehension of the presented task had to be filled in within the application after the experimental trials. Finally, all participants filled in a paper-based form of the SDS-17 questionnaire assessing social desirability.
