# Supplementary material for: How does the method change what we measure? Comparing virtual reality and text-based surveys for the assessment of moral decisions in traffic dilemmas
Source: PLoS One. 2019 Oct 9;14(10):e0223108. doi: 10.1371/journal.pone.0223108 (PMC6785059; doi:10.1371/journal.pone.0223108)
Supplement: S3 Appendix — (PDF) [file pone.0223108.s004.pdf]

	trials	switches	trials with switches	h. vs h. trials	error rate	RT mean (SD)	% RT under VR limit
VR naturalistic	860	945	503 (58.5%)	295	0.54%	1.42s (1.02s)	-
VR text-based	860	586	450 (52.3%)	278	0%	1.65s (0.79s)	-
Desktop natural.	840	-	-	274	0%	2.60s (3.08s)	82.4%
Desktop text-b.	840	-	-	289	0.64%	0.64s (3.85s)	72.2%
total	3400	-	-	1136	0.29%	-	-

Table 1. Study 1. Left to right: Total number of trials, total number of lane switches, number of trials in which lane switches were performed, number of trials with humans in either lane, error rate estimate (rate of ”saving” the empty lane), response time means (VR: only taking trials with lane switches into account), percentage of responses within the time limit of the corresponding VR conditions (naturalistic: 4.0s, text-based: 4.4s).

	trials	switches	trials with switches	h. vs h. trials	error rate	RT mean (SD)
VR naturalistic slow	651	645	363 (55.8%)	465	0%	1.62s (1.11s)
VR text-based slow	651	363	328 (50.4%)	466	0%	1.55s (0.73s)
VR naturalistic fast	651	526	371 (57.0%)	468	1.11%	0.61s (0.29s)
VR text-based fast	651	396	348 (53.5%)	474	1.70%	1.02s (0.25s)
total	2604	1930	1410 (54.1%)	1873	0.55%	-

Table 2. Study 2. Left to right: Total number of trials, total number of lane switches, number of trials in which lane switches were performed, number of trials with humans in either lane, error rate estimate (rate of ”saving” the empty lane), response time means (only taking trials with lane switches into account).

Supporting information

S3 Appendix: Trials and error rates. **Study 1.** A total of 3400 trials were analyzed in the first study, i.e., between 840 and 860 in each of the four conditions. Of these, 1136 trials (274 to 295 per condition) were direct juxtapositions of humans. In order to estimate the frequency of erroneous trials in our data, we asked participants in a post-hoc questionnaire to recall the number of errors they had made during their trials (e.g., accidentally hitting the wrong button). A total of 30 errors were reported for 3400 trials (0.9%). Furthermore, we took the rate of hitting an obstacle when the other lane was empty as a second measure, which was observed in 2 out of 688 trails (0.3%). We take from these figures that errors in the response are unlikely to have a strong impact on the results. Moreover, we found animals to be saved over humans in only 17 out of 1354 trials (1.3%). Since this rate is very close to the error rate estimators, it seems plausible that species is an almost trivial factor within the participants' demographic. We, therefore, decided to focus on trials with humans in either lane for the behavioral analysis of study 1.

Study 2. In the second study, a total of 2604 trials (651 per condition) was recorded, 1873 of which were juxtapositions of humans. As an estimation of the error rate, we again used the rate of trials in which a human was hit despite the other lane being empty. This never occurred in the slow conditions, but happened in 1.7% and 1.1% of trials in the fast text-based and fast naturalistic condition, respectively. Note that since the task of correctly identifying age and gender of the obstacles is arguably harder than identifying the mere existence of an obstacle, misinterpretations of the scene could have caused the true error rate to be higher than the estimator under severe time constraints.
