# Supplementary material for: How does the method change what we measure? Comparing virtual reality and text-based surveys for the assessment of moral decisions in traffic dilemmas
Source: PLoS One. 2019 Oct 9;14(10):e0223108. doi: 10.1371/journal.pone.0223108 (PMC6785059; doi:10.1371/journal.pone.0223108)
Supplement: S4 Appendix — (PDF) [file pone.0223108.s005.pdf]

Supporting information

S4 Appendix: Bayesian logistic regression model specifications. Study 1. The dependent variable for all models was whether subjects choose the left lane (1) or the right (0). For the first study, we used as predictors the intercept (*1*; coding for a lane-bias towards the left lane), the lane the participants were in at the moment of the obstacles' visibility onset (*omission.bias*; coding for a bias to stay in the lane), the configuration of obstacles in the two lanes, as well as the *modality* and *abstraction* level of the respective experimental condition and their interactions. Note that there was no default choice indicated in the desktop conditions, and thus no omission bias or effect of the modality on an omission bias can be calculated. The configuration of obstacles in the two lanes was analyzed similar to Bradley-Terry-Luce models [1] and was coded as follows: *gender.bias* is 0 if both obstacles have the same gender, .5 if the left obstacle is male and the right obstacle is female, and $-.5$ vice versa. *young.bias* is 0 if both obstacles are children, .5 if the right obstacle is a child but the left obstacle is not, and $-.5$ vice versa. *elderly.bias* is the corresponding equivalent for the elderly. The adult obstacles weren't explicitly modeled, as it would result in an over-specification of the model. The model can thus be understood as fixing the model parameters for adults at 0 and modeling the other two age groups relative to this. *Modality* (VR/ desktop) and *abstraction* (text-based/ naturalistic) used effect coding, i.e., the previously specified effects do not represent the effect at one specific condition, but at the average over the four conditions. The predictions for any particular condition are obtained by adding or subtracting one half of the respective modality and abstraction parameters. All models used weakly regularizing priors. For parameter means, we used Normal($\mu = 0, \sigma = 3$), for the variance of the distribution of parameters by subject we used Cauchy($x_0 = 0, \gamma = 1$), and for co-variance matrices we used LKJ($\eta = 2$). For all analysis we made use of the BRMs package [2,3] using the NUTS Hamiltonian Monte Carlo algorithm [4,5]. We used 8000 samples for each chain including 2000 warm up samples with 4 independent MCMC chains. We report 95% credibility intervals of the posterior and the mean posterior value. Bayes factors were calculated using the Savage-Dickey density ratio method as implemented in the brms package. We interpret Bayes factors between 1/3 and 3 as mostly inconclusive, Bayes factors above 10 as strong evidence. The model for study 1 was specified as follows:

$$\begin{aligned}\text{choice.left}_{\text{S1}} \sim\ & 0 + \text{Intercept}\\ & +(\text{omission.bias} + \text{gender.bias} + \text{young.bias} + \text{elderly.bias}) * \text{modality} * \text{abstraction}\\ & +(0 + \text{Intercept} + (\text{omission.bias} + \text{gender.bias} + \text{young.bias} + \text{elderly.bias}) * \text{modality} * \text{abstraction}|\text{subj.idx})\end{aligned}$$

Study 2. The model used in the second study is based on that of the first study, with the following changes: *Speed* replaces *modality* and was coded in the same $-0.5/0.5$ scheme. In addition to the features of the portrayed situation and the features of the experimental condition, we included features of the individual subjects in the model: *subj.gender* is the gender of the subject in the trial (using effect coding, i.e., ± 0.5), *subj.age* is their age (variable centered, reported effects are effects per year of age), *subj.gamehrs* is their reported weekly average of hours spent playing video games (variable centered, reported effects are effects per one hour of weekly playtime), and *subj.sds17* is the score of their SDS-17 questionnaire (variable centered, reported effects are effects per one score in the test). The model was specified as follows:

$$
\begin{aligned}
\text{choice.left}_{S2} \sim\ & 0 + \text{Intercept} \\
& +(\text{omission.bias} + \text{gender.bias} + \text{young.bias} + \text{elderly.bias}) * \text{speed} * \text{abstraction} \\
& +(0 + \text{Intercept} + (\text{omission.bias} + \text{gender.bias} + \text{young.bias} + \text{elderly.bias}) * \text{speed} * \text{abstraction} | \text{subj.idx}) \\
& +(\text{omission.bias} + \text{gender.bias} + \text{young.bias} + \text{elderly.bias}) * \text{subj.gender} \\
& +(\text{omission.bias} + \text{gender.bias} + \text{young.bias} + \text{elderly.bias}) * \text{subj.age} \\
& +(\text{omission.bias} + \text{gender.bias} + \text{young.bias} + \text{elderly.bias}) * \text{subj.gamehrs} \\
& +(\text{omission.bias} + \text{gender.bias} + \text{young.bias} + \text{elderly.bias}) * \text{subj.sds17}
\end{aligned}
$$

References

1. Bradley RA, Terry ME. Rank analysis of incomplete block designs: I. The method of paired comparisons. Biometrika. 1952;39(3/4):324–345.

2. Bürkner PC. brms: An R Package for Bayesian Multilevel Models Using Stan. Journal of Statistical Software. 2017;80(1):1–28. doi:10.18637/jss.v080.i01.

3. Bürkner PC. Advanced Bayesian Multilevel Modeling with the R Package brms. The R Journal. 2018;10(1):395–411.

4. Hoffman MD, Gelman A. The No-U-Turn sampler: adaptively setting path lengths in Hamiltonian Monte Carlo. Journal of Machine Learning Research. 2014;15(1):1593–1623.

5. Carpenter B, Gelman A, Hoffman MD, Lee D, Goodrich B, Betancourt M, et al. Stan: A probabilistic programming language. Journal of statistical software. 2017;76(1).
