# Supplementary material for: How does the method change what we measure? Comparing virtual reality and text-based surveys for the assessment of moral decisions in traffic dilemmas
Source: PLoS One. 2019 Oct 9;14(10):e0223108. doi: 10.1371/journal.pone.0223108 (PMC6785059; doi:10.1371/journal.pone.0223108)
Supplement: S5 Appendix — (PDF) [file pone.0223108.s006.pdf]

Supporting information

S5 Appendix: Regression coefficients. **Study 1.** All $\hat{R}$ values were below 1.1 indicating convergence. Due to the high number of predictors only a random selection of predictors was visually inspected for convergence. We noticed that in study one, the second level standard deviation marginal density for the effect "elderly.bias" was bimodal (but not the fixed coefficient). None of the correlations on the population level showed indication for an effect. It is very likely that our model was too complex to estimate such correlations reliably given our sample size and the lkj-prior takes over. Table 1 reports the fixed effects conditional on the population level. The main effects of the coefficients are all very large, indicating a binary decision by most subjects.

**parameter**	**mean**	**SD**	**2.5%**	**97.5%**	BF_{H0}
lane.bias	-0.17	0.17	-0.51	0.16	11.07
omission.bias	1.44	0.56	0.39	2.58	0.14
gender.bias	3.01	0.39	2.30	3.82	0.00
young.bias	9.51	1.26	7.24	12.16	-0.00
elderly.bias	-7.41	1.04	-9.64	-5.55	0.00
lane.bias:abstraction	0.38	0.30	-0.21	0.96	4.59
omission.bias:abstraction	-1.28	0.82	-2.92	0.31	1.10
gender.bias:abstraction	0.29	0.68	-1.03	1.63	4.08
young.bias:abstraction	-0.05	1.38	-2.80	2.61	2.24
elderly.bias:abstraction	1.84	1.10	-0.26	4.04	0.69
lane.bias:modality	0.16	0.33	-0.50	0.82	8.29
gender.bias:modality	-0.69	0.69	-2.05	0.66	2.68
young.bias:modality	1.05	1.71	-2.34	4.41	1.46
elderly.bias:modality	1.98	1.51	-0.93	5.04	0.85
lane.bias:modality:abstraction	0.30	0.59	-0.86	1.47	4.60
gender.bias:modality:abstraction	0.80	1.26	-1.67	3.28	2.00
young.bias:modality:abstraction	-0.22	2.09	-4.32	3.94	1.42
elderly.bias:modality:abstraction	1.19	1.82	-2.33	4.78	1.34

Table 1. Model coefficients with standard deviation and credible intervals for study 1.

Study 2. The results of study two, shown in Table 2 are very similar. No bimodality was found in the marginal posterior densities.

References

**parameter**	**mean**	**SD**	**2.5%**	**97.5%**	BF_{H0}
lane.bias	0.06	0.14	-0.21	0.33	21.31
omission.bias	0.61	0.34	-0.07	1.30	1.70
gender.bias	2.61	0.38	1.90	3.41	0.00
young.bias	8.11	0.96	6.37	10.15	0.00
elderly.bias	-6.88	0.77	-8.51	-5.49	-0.00
lane.bias:speed	0.32	0.23	-0.12	0.78	4.90
omission.bias:speed	-0.21	0.46	-1.11	0.72	5.95
gender.bias:speed	-0.95	0.53	-2.00	0.09	1.09
young.bias:speed	-3.05	0.85	-4.78	-1.45	0.00
elderly.bias:speed	2.51	0.90	0.79	4.33	0.08
lane.bias:abstraction	-0.03	0.27	-0.55	0.49	11.38
omission.bias:abstraction	-0.59	0.47	-1.52	0.30	2.89
gender.bias:abstraction	-0.34	0.52	-1.36	0.67	4.76
young.bias:abstraction	1.81	0.86	0.17	3.54	0.37
elderly.bias:abstraction	4.57	0.86	2.97	6.35	0.00
lane.bias:speed:abstraction	-0.04	0.45	-0.90	0.83	6.94
omission.bias:speed:abstraction	-0.14	0.86	-1.80	1.54	3.43
gender.bias:speed:abstraction	-0.33	0.97	-2.24	1.57	2.95
young.bias:speed:abstraction	1.71	1.46	-1.16	4.57	1.08
elderly.bias:speed:abstraction	0.26	1.40	-2.50	3.02	2.17
lane.bias:subj.gender	-0.03	0.31	-0.63	0.59	9.84
omission.bias:subj.gender	0.49	0.76	-0.98	2.00	3.31
gender.bias:subj.gender	-0.38	0.76	-1.89	1.12	3.63
young.bias:subj.gender	-0.43	1.58	-3.54	2.67	1.87
elderly.bias:subj.gender	-1.81	1.21	-4.21	0.58	0.78
lane.bias:subj.age	0.04	0.04	-0.03	0.12	41.57
omission.bias:subj.age	-0.02	0.09	-0.21	0.17	32.29
gender.bias:subj.age	0.13	0.10	-0.06	0.33	13.38
young.bias:subj.age	-0.13	0.22	-0.55	0.31	12.01
elderly.bias:subj.age	-0.11	0.17	-0.45	0.21	15.11
lane.bias:subj.sds17	0.04	0.05	-0.05	0.14	43.02
omission.bias:subj.sds17	0.18	0.12	-0.05	0.42	7.28
gender.bias:subj.sds17	0.16	0.12	-0.08	0.40	10.39
young.bias:subj.sds17	-0.06	0.28	-0.63	0.49	10.94
elderly.bias:subj.sds17	-0.10	0.20	-0.49	0.29	13.74
lane.bias:subj.gamehrs	-0.01	0.03	-0.08	0.05	90.07
omission.bias:subj.gamehrs	-0.01	0.08	-0.17	0.14	39.72
gender.bias:subj.gamehrs	0.05	0.08	-0.11	0.21	32.72
young.bias:subj.gamehrs	0.02	0.18	-0.33	0.38	17.48
elderly.bias:subj.gamehrs	0.01	0.13	-0.24	0.26	23.99

Table 2. Model coefficients with standard deviation and credible intervals for study 2.
